# Changes in auditory perceptions and cortex resulting from hearing recovery after extended congenital unilateral hearing loss

**DOI:** 10.3389/fnsys.2013.00108

**Published:** 2013-12-13

**Authors:** Jill B. Firszt, Ruth M. Reeder, Timothy A. Holden, Harold Burton, Richard A. Chole

**Affiliations:** ^1^Department of Otolaryngology-Head and Neck Surgery, Washington University School of MedicineSt. Louis, MO, USA; ^2^Department of Anatomy and Neurobiology, Washington University School of MedicineSt. Louis, MO, USA; ^3^Department of Radiology, Washington University School of MedicineSt. Louis, MO, USA; ^4^Department of Developmental Biology, Washington University School of MedicineSt. Louis, MO, USA

**Keywords:** unilateral hearing loss, congenital, conductive, stapedotomy, brain imaging, sound localization, speech recognition

## Abstract

Monaural hearing induces auditory system reorganization. Imbalanced input also degrades time-intensity cues for sound localization and signal segregation for listening in noise. While there have been studies of bilateral auditory deprivation and later hearing restoration (e.g., cochlear implants), less is known about unilateral auditory deprivation and subsequent hearing improvement. We investigated effects of long-term congenital unilateral hearing loss on localization, speech understanding, and cortical organization following hearing recovery. Hearing in the congenitally affected ear of a 41 year old female improved significantly after stapedotomy and reconstruction. Pre-operative hearing threshold levels showed unilateral, mixed, moderately-severe to profound hearing loss. The contralateral ear had hearing threshold levels within normal limits. Testing was completed prior to, and 3 and 9 months after surgery. Measurements were of sound localization with intensity-roved stimuli and speech recognition in various noise conditions. We also evoked magnetic resonance signals with monaural stimulation to the unaffected ear. Activation magnitudes were determined in core, belt, and parabelt auditory cortex regions via an interrupted single event design. Hearing improvement following 40 years of congenital unilateral hearing loss resulted in substantially improved sound localization and speech recognition in noise. Auditory cortex also reorganized. Contralateral auditory cortex responses were increased after hearing recovery and the extent of activated cortex was bilateral, including a greater portion of the posterior superior temporal plane. Thus, prolonged predominant monaural stimulation did not prevent auditory system changes consequent to restored binaural hearing. Results support future research of unilateral auditory deprivation effects and plasticity, with consideration for length of deprivation, age at hearing correction and degree and type of hearing loss.

## Introduction

Monaural hearing induces auditory system reorganization. This occurs in cases of unilateral sensorineural hearing loss, where there is sensory (inner ear) or neural dysfunction, as well as losses in the conductive pathway between the outer and inner ear. In animals, unilateral conductive hearing loss (UCHL) results in structural and functional changes within the auditory system. For example, on the UCHL side in rats, the size of neurons in the anteroventral cochlear nucleus was smaller (Coleman and O’Connor, 1979) and binaural interactions were absent in the inferior colliculus (Silverman and Clopton, 1977). Likewise in cats, UCHL reduced the inhibition from the ipsilateral ear on neurons in the inferior colliculus when the contralateral ear was deprived of sound (Moore and Irvine, 1981). Additionally, UCHL leads to decreased 2-deoxyglucose uptake bilaterally in the higher auditory medial and lateral superior olive nuclei even in silence, indicating decreased neuronal activity (Tucci et al., 2001). Interestingly, in a number of these studies, bilateral conductive hearing loss had little to no effect on neuronal size or binaural interactions, with normal maintenance of contralateral and ipsilateral projections. Together, these studies suggest that modifications in the balance of afferent activity alter binaural interactions and auditory system structures.

Imbalanced input from UCHL also degrades signal segregation for listening in noise and time-intensity cues for sound localization in humans (Wilmington et al., 1994; Gray et al., 2009). Amongst tasks requiring binaural processing, UCHL participants had lower interaural temporal difference limens, required higher intensities in the affected ear to perceive balanced loudness, had lower speech recognition in noise, and for masking level differences (MLDs), had signal detection affected by lower amplitude noise levels (Hall and Grose, 1993; Wilmington et al., 1994; Gray et al., 2009). Self-assessment questionnaires that probe hearing in different listening environments indicated numerous hearing difficulties in those with congenital UCHL (Priwin et al., 2007). While there have been studies of bilateral sensorineural auditory deprivation and later hearing restoration from implantable devices (e.g., cochlear implants), much less is known about unilateral auditory deprivation from conductive hearing loss and subsequent hearing improvement.

Effects of early abnormal auditory experience and later recovery have been reported in several animal studies. For example, UCHL created by plugging one ear in young owls initially altered localization abilities but abilities then recovered, that is, the owls made use of abnormal cues to accurately localize (Knudsen et al., 1984a,b). After the earplug was removed, these owls again made localization errors but after some weeks they regained accuracy. However, localization performance did not recover when ear plugging occurred in older owls or even in younger owls when the plug remained in place beyond a few weeks. Thus, the ability to recover was affected by the age at which UCHL occurred as well as the age at which hearing was restored. While it is well known that unilateral deprivation of the visual system early in life results in permanent impairment of binocular vision (Hubel and Wiesel, 1970), less is known about the developing auditory system’s ability to recover binaural abilities. Undefined in humans is a sensitive period for binaural hearing, where behavior can adapt to abnormal experience and develop accurate abilities, and a critical period, after which the ability to adapt to altered (including restored) hearing is greatly diminished or nonexistent.

Binaural performance improved in some individuals who had UCHL correction when the loss was acquired after maturation (Hausler et al., 1983; Hall and Derlacki, 1986, 1988). Hall and colleagues showed, using measures of MLDs, that reduced binaural abilities may continue after restoration of hearing in adults with otosclerosis (Hall and Derlacki, 1986, 1988; Hall et al., 1990). However, in a later longitudinal study of adults with otosclerosis, MLDs returned to normal in many participants when tested a year after hearing restoration (Hall and Grose, 1993). Otosclerosis is typically diagnosed in young to middle adulthood and the hearing loss is often progressive in nature (and primarily conductive), thus these individuals had several years of normal hearing (NH) prior to hearing loss onset.

Congenital conductive hearing loss may occur for reasons of atresia or middle ear anomalies such as a fused malleus and incus, or fixation of the stapes. Degree of hearing loss and the presence or absence of other inner ear deficits varies with the abnormality, but can result in 60 dB of hearing loss from the conductive aspect alone. Diagnosis of hearing loss and treatment to improve hearing often occurs in childhood. When left untreated into adulthood, individuals will have extended periods of auditory deprivation.

A prior study assessed binaural abilities in patients aged 6–33 years, before and after surgery to correct congenital UCHL (Wilmington et al., 1994). Measures included interaural temporal difference limens, MLDs, sound localization and speech recognition in noise. Post-surgery binaural improvements were significant for some individuals and some tasks but not all; sound localization and speech recognition continued to be difficult especially when noise was towards the NH ear and the restored ear was relied upon for speech understanding. Abnormal auditory experience in early life may affect later binaural abilities; however, the nature and impact of these interactions are not entirely clear.

In the current case study, we investigated the effects of long-term congenital, unilateral mixed (conductive and sensorineural) hearing loss on sound localization, speech understanding, and cortical organization, both before and after the conductive component of the hearing loss was corrected in adulthood.

## Materials and Methods

Written informed consent was obtained from the participant in accordance with the Declaration of Helsinki and guidelines approved by the Human Research Protection Office at Washington University School of Medicine (WUSM).

### Participant

**Figure 1:**
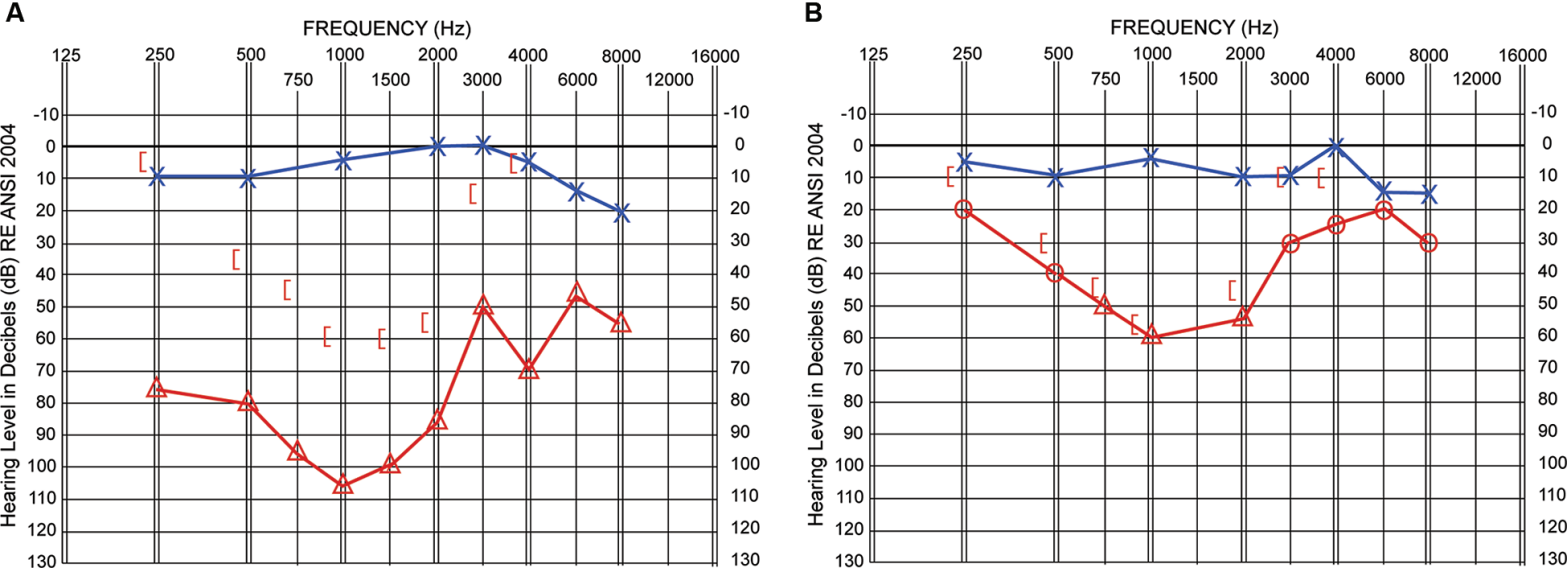
**Hearing thresholds in decibels (dB; *y*-axis) are shown as a function of frequency (Hz; *x*-axis).** Pre-surgical results in Panel **A** and 3-month post-surgical results in Panel **B**. Air-conduction thresholds via TDH headphones (Panel **A**) or insert earphones (Panel **B**) are indicated with blue Xs for the left ear and red triangles or circles for the right ear. Circles indicate unmasked right ear thresholds and triangles indicate thresholds obtained with masking noise presented to the better hearing left ear. Masked bone-conduction thresholds for the right ear are indicated with red left-facing brackets.

The participant (P1) was a 41 year old female who had a history of unilateral hearing loss in the right ear that had been present since early childhood (probably since birth). Family history included a father and paternal grandmother with mixed unilateral hearing loss. P1’s father had successful stapes surgery in adulthood as a treatment for otosclerosis induced hearing loss. P1 and her family had believed P1’s hearing loss to be sensorineural and non-correctable. A hearing aid was fitted around age 5 but discontinued after a brief trial due to lack of benefit. In adulthood, the participant had a comprehensive audiological evaluation that diagnosed the loss as mixed. P1 consulted an otolaryngologist to discuss treatment options. Audiological air-conduction results (see Figure 1A) for the right ear (red triangles) indicated a severe hearing loss at 0.25 and 0.5 kHz, dropping to a profound hearing loss at 1 kHz and rising to a moderate to moderately-severe hearing loss from 3 to 8 kHz. Bone-conduction results (red brackets) identified the loss as mixed with a conductive component (30–70 dB) at all frequencies and an additional sensorineural component from 0.5 to 2 kHz. The difference between the air- and bone-conduction thresholds identifies the conductive component of the participant’s hearing loss, that is, the portion of the loss that could potentially be corrected through surgery. Hearing levels were normal in the unaffected left ear (blue symbols) resulting in a large hearing asymmetry between ears. With this level of asymmetry and the mixed nature of the hearing loss in the affected ear, audiological masking approaches become more complicated due to potential cross masking effects. One-third octave narrow bands of masking noise (centered at the test frequency) were presented to the unaffected ear to isolate the affected ear during testing. Testing was completed with TDH-49 headphones with 40–60 dB of interaural attenuation. Although a 15 dB plateau was achieved at each frequency (increases in masking level at the unaffected ear did not increase the patient’s response level in the affected ear), high levels of masking noise to the unaffected ear could possibly have been detected by the cochlea of the affected ear via bone conduction while attempting to identify accurate air-conduction thresholds of the affected ear.

Pre-operative, high resolution CT scanning revealed a slightly thickened stapes footplate with no other abnormalities suggestive of otosclerosis. The participant underwent a laser stapedotomy procedure with prosthetic reconstruction completed under general anesthesia through an aural speculum. This procedure is designed to restore mobility to the middle ear bones for improved sound conduction. A tympanomeatal flap was raised and the middle ear entered. The malleus and incus were mobile and the stapes firmly fixed in the oval window consistent with a congenitally fixed stapes. A CO_2_ laser was used to make a stapedotomy in the center of the stapes footplate and a Teflon piston was placed and connected to the incus. The meatal flap was returned into position.

### Test Measures

All testing occurred in double-walled sound booths with the participant comfortably seated. All test stimuli were from recordings and individually calibrated for the presentation equipment. Study measures were conducted pre-surgery and 3 and 9 months post-surgery.

Localization was measured with a roving-source Consonant-Vowel-Consonant (CNC) word task (Potts et al., 2009; Firszt et al., 2012a). American English Melbourne CNC words (Skinner et al., 2006) were presented randomly from 15 loudspeakers (10° apart and numbered 1–15) along a horizontal plane and 140° arc at 60 dB SPL (±3 dB). Each test administration included 100 words, presented randomly from each of the 10 active loudspeakers with the carrier “Ready”. The participant sat approximately three feet in front of the center loudspeaker, was unaware that five loudspeakers were inactive (#2, 4, 8, 12, 14), and was instructed to face the center loudspeaker between each presentation but was allowed head turns during each carrier-word presentation. Head turns were allowed since it more accurately simulates talker localization in conversational settings. After each presentation, the participant repeated the word and indicated the source speaker number. For localization, a root mean square (RMS) error score was calculated as the mean target-response difference, irrespective of error direction. The task was administered twice and scores from each administration averaged.

Sentence understanding in noise was evaluated with the Hearing In Noise Test (HINT; Nilsson et al., 1994) in the *R*-Space (Revit et al., 2002; Compton-Conley et al., 2004). The *R*-Space consists of eight loudspeakers equally spaced in a 360° array with each loudspeaker 24 inches from the center of the participant’s head. All loudspeakers presented a recording of diffuse restaurant noise (60 dB SPL), replicating a challenging real-life listening situation. Two 20 sentence lists were administered and an average signal-to-noise ratio (SNR) for 50% accuracy (SNR-50) obtained. Sentences were presented from the front loudspeaker beginning at a +6 dB SNR and adapted to be easier or more difficult based on the participant’s responses. An average of the final 17 SNRs resulted in an SNR-50 for each list.

An adaptive speech reception threshold (SRT) psychoacoustic task resulted in speech thresholds for quiet and nine competing noise conditions (modified from the task described in Litovsky (2005) and Johnstone and Litovsky (2006)). SRT determinations were for test spondees (two-syllable words with equal stress on both syllables) spoken by a male talker and always presented from a loudspeaker facing the front of the seated participant. Testing occurred with noise emanating from one of three loudspeakers each placed 1.5 m from the participant: directly in front, 90° to the right, or 90° to the left. There were three noise types: multi-talker babble (MTB) and two types of single-talker noise (a female talker and a male talker each presenting Harvard IEEE sentences). Initial spondee presentations were at 60 dB SPL for each noise type and source location. A four-alternative forced-choice task, without feedback, determined subsequent presentation levels that continued through four reversals using an adaptive paradigm based on participant responses. Noise conditions varied randomly and were tracked independently, resulting in an SRT (average of the last three reversals) for each noise type and loudspeaker location (e.g., front, left, or right for MTB, female talker, and male talker noise).

A psychoacoustic measure determined the random spectrogram sound (RSS) that was most different from two others during each trial. RSS are noise-like stimuli created with independent control of temporal or spectral sound parameters, but with all stimuli based on summation across the same 6-octave bandwidth (250–16000 Hz) and with matching intensity and durations (Schönwiesner et al., 2005; Burton et al., 2012). Spectral RSS differed by dividing the bandwidth into 3–16 spectral bands and changing between bands at a fixed temporal rate of 3 Hz. Temporal RSS differed by changing between three spectral bands at temporal rates between 8 and 30 Hz. Spectral RSS had greater complexity with more spectral bands and temporal RSS were more complex with higher temporal rates. In a three-interval, three-alternative, forced-choice, “odd-man out” paradigm, one of the stimuli, identified as the “target” differed in complexity from two “standard” stimuli. “Standard” sounds always had the same RSS complexity, but differed in constituent random fields or in amplitude modulations. The manipulated variable was minimum detectable changes (JND) in spectral or temporal complexity.

For spectral RSS, the number of spectral regions of the “standard” was 16, while the spectral regions for the “target” varied. The initial “target” had three spectral regions. After three consecutive correct responses, the “target” spectral regions increased with a “step-size” of three until an error occurred. After the first reversal, the “step-size” decreased to two spectral regions, and after three reversals, the “step-size” decreased to one spectral region. For temporal RSS, the temporal rate of the “standard” was 30 Hz, while the temporal rate varied for the “target” stimuli. The initial “target” temporal rate was 8 Hz. After three consecutive correct responses, the “target” temporal rate increased to 14 Hz. The same 6 Hz “step-size” in temporal rate repeated until an error occurred. After the first reversal, the “step-size” decreased to 3 Hz, and after three reversals, the “step-size” decreased to 1 Hz. After eight reversals, the “mean of target” values of the last four reversals provided an estimate of JNDs for spectral or temporal complexity. Feedback was provided for correct responses and each of four test runs concluded with eight reversals. Each of the JNDs for spectral and temporal RSS complexity was an average of the last four reversals.

A questionnaire also evaluated the participant’s perception of listening function. The Speech, Spatial and Qualities of Hearing scale (SSQ; Gatehouse and Noble, 2004) probes three listening domains (14–19 questions each). The Speech domain probes speech recognition in a variety of listening environments and a range of talker visibility. The Spatial domain examines awareness of sound direction, distance and movement. In the Qualities domain, respondents indicate sound naturalness, listening effort and the ability to segregate multiple sounds. Each item is ranked on a scale from 0 (least ability) to 10 (most ability). Individual SSQ items are grouped by processing demands to obtain 10 subscale scores (Gatehouse and Akeroyd, 2006; Dwyer et al., 2013), four subscales within the Speech domain, two within the Spatial domain, and four within the Qualities domain.

Previously described protocols (Burton et al., 2012, 2013) were used to perform functional magnetic resonance imaging (fMRI), preprocess the images, and analyze blood oxygen level-dependent (BOLD) responses to auditory stimulation of the unaffected left ear. BOLD responses to RSS stimuli of 2 s duration were recorded at three times: pre-surgery and at 3 and 9 months post-surgery. Presentation times for RSS stimuli varied during 9-s silent intervals in 11-s volume acquisitions, which enabled capture of BOLD at different stimulus delays with respect to the echo-planar images (EPI) in this interrupted single event design (Belin et al., 1999). Subsequent assembly of BOLD amplitudes relative to baseline (% change in response) at stimulus delays from 2 to 9 s prior to the EPI reconstructed an average BOLD response time course. The average was from 24 trials for each stimulus to EPI delay time. Separately for each imaging session, *F*-tests per voxel evaluated whether the variance of evoked BOLD responses was greater than variance due to baseline noise. *F*-statistics were transformed to equally probable *z*-scores (*F*-*Z*stats). The significance of *F*-*Z*stats was determined after multiple comparison corrections based on Monte Carlo simulations (Forman et al., 1995) and with a correction threshold of *z* = 4.0 across 12 face-connected voxels and for *p* = 0.05.

The distribution of volume-based *F*-*Z*stats from each imaging session was superimposed on coronal slices through the patient’s brain. Additionally, BOLD response time courses were extracted from regions of interest for peak *F*-*Z*stats identified with an automated search (Kerr et al., 2004). After registering the *F*-*Z*stats to the PALS-B12 surface-based atlas (Van Essen, 2005; Van Essen and Dierker, 2007), the activated auditory cortical fields were evaluated as previously described (Burton et al., 2012). Thus, the analyses evaluated activity in core primary auditory (Te1), planum temporale (Te2), and planum polare (Te3).

## Results

Figure 1B shows post-surgical audiometric thresholds as a function of frequency. (Results from the 3-month evaluation are shown. Note that the 9-month results were equivalent.) As expected, thresholds for the NH or left ear (blue symbols) were unchanged compared to pre-surgical results. The red line connects post-surgical air-conduction thresholds (obtained with insert earphones having approximately 75–90 dB interaural attenuation). Open circles indicate unmasked thresholds and open triangles indicate thresholds obtained with the unaffected ear masked. Post-surgical bone-conduction thresholds are indicated by red brackets. Air-conduction thresholds in the right ear improved 35–45 dB through the low and mid frequencies and 5–40 dB in the high frequencies. Surgery successfully eliminated the hearing loss conductive component from 0.5–3 kHz and most of the conductive component at 0.25 and 4 kHz.

**Figure 2:**
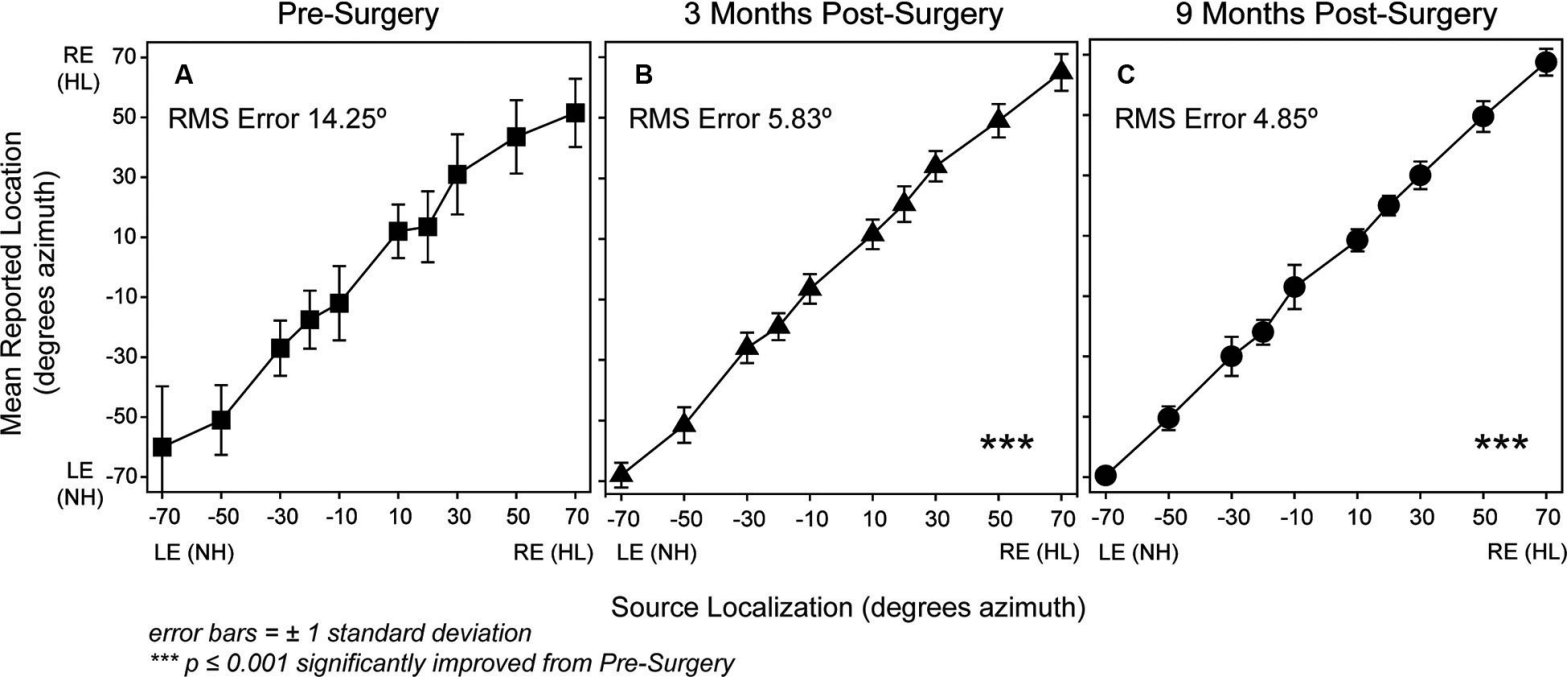
**P1’s localization responses are plotted for each test interval (Panel A, pre-surgery, square symbols; Panel B, 3 months post-surgery, triangle symbols; Panel C, 9 months post-surgery, circle symbols).** The *x*-axis shows the sound-source loudspeaker locations in degrees azimuth that range from −70 degrees toward the NH ear side at axis’ left to +70 degrees toward the hearing-impaired ear side at the axis’ right. The *y*-axis shows the possible reported response loudspeaker locations in degrees azimuth that range from −70 degrees toward the NH ear side at the bottom of the axis to +70 degrees toward the hearing-impaired ear side at the top of the axis. Symbols indicate mean responses for each active sound-source location and error bars represent ±1 standard deviation. Mean RMS Error in degrees is noted within the plot for each test interval. Asterisks in Panels **B** and **C** indicated significantly improved localization results compared to pre-surgery (*p* < 0.001).

Figure 2 shows localization results. For each plot, the location of the loudspeaker source (in degrees azimuth) is indicated along the *x*-axis and of the reported loudspeaker along the *y*-axis. Means and standard deviations of reported loudspeakers are plotted for each source loudspeaker location for pre-surgery and post-surgery test intervals of 3 and 9 months, respectively in Panels A to C. Correct identification of all presentations results in a straight diagonal line from the lower left- to the upper right corner. Each panel also includes the RMS error score for that test interval. A robust (Sandwich estimator) regression analysis method (with a Bonferroni adjustment for multiple comparisons) identified a significant effect of test interval [*F*(2,594) = 11.97, *p* < 0.003] and follow-up comparisons indicated significantly improved localization compared to baseline at 3 months [*F*(1,549) = 17.26, *p* < 0.003] and at 9 months [*F*(1,594) = 23.70, *p* < 0.001]. The improvement at 9 months compared to 3 months was not statistically significant (*p* > 0.05).

**Figure 3:**
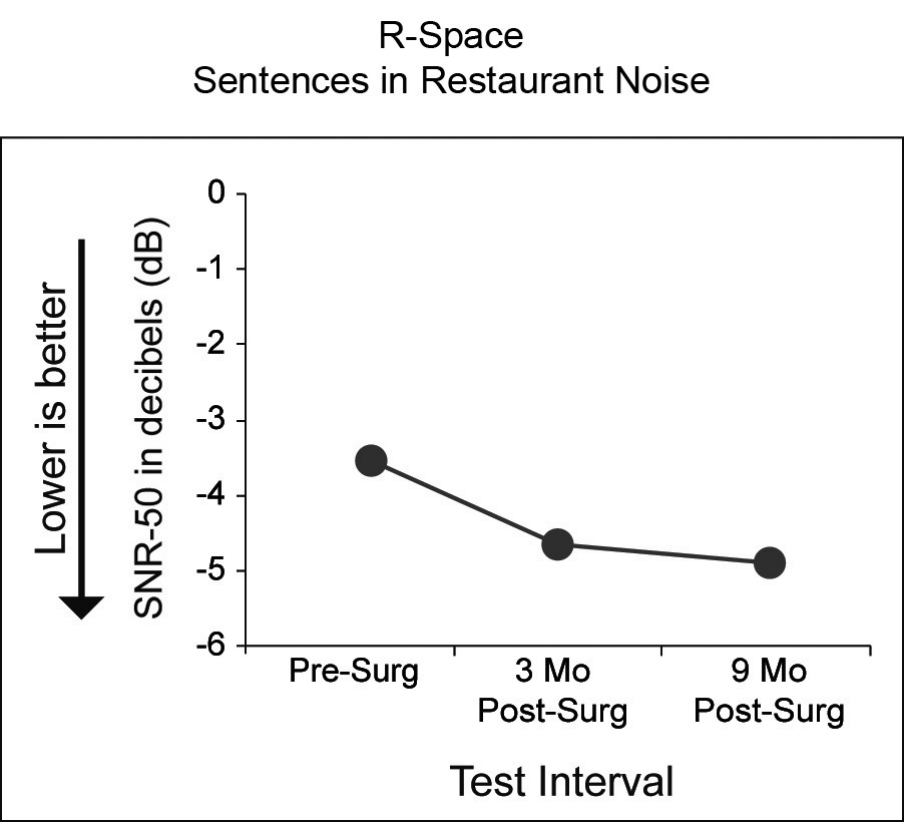
**Results of sentence understanding in the R-Space using restaurant noise is shown for the three test intervals (*x*-axis).** Black circles indicate P1’s SNR-50 scores in dB. Lower SNRs reflect ability to understand sentences in the presence of higher noise levels (e.g., an SNR of −4 dB indicates the restaurant noise was 4 dB louder than the target sentence).

Figure 3 displays results for *R*-Space sentence understanding in the presence of restaurant noise. The three test intervals are indicated along the *x*-axis and SNR score along the *y*-axis. Note that for this measure lower scores indicate better performance. Pre-surgery, P1 had 50% accuracy for sentences presented 3.5 dB softer than the surrounding restaurant noise (60 dB SPL). By 9 months post-surgery, P1 was able to understand sentences at even softer levels (4.9 dB softer than the restaurant noise). P1’s performance improved (1.4 dB) from pre-surgery to the 9-month test interval. The 95% confidence interval for NH adults and noise from the front is ±1.2 dB (Nilsson et al., 1995).

**Figure 4:**
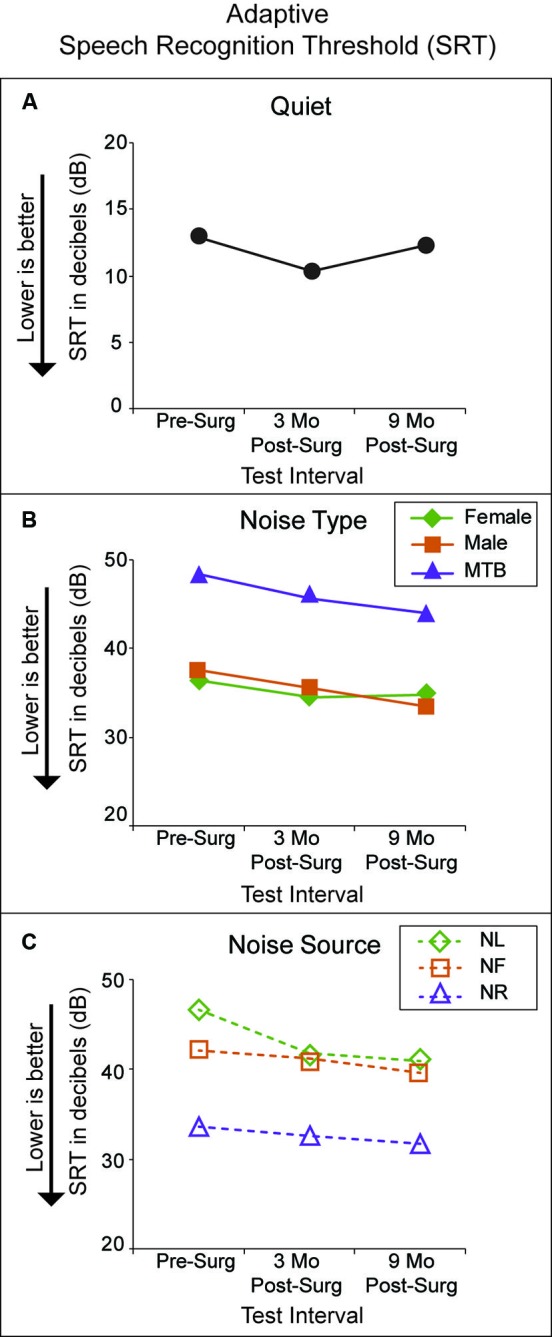
**Adaptive SRT scores in dB (*y*-axis) are plotted for each test interval (*x*-axis).** Lower SRTs reflect ability to hear speech at softer levels. Panel **A** plots results in quiet (black circles). Panel **B** plots results in noise by noise type (female talker with green diamonds; male talker with orange squares; MTB with purple triangles). Panel **C** plots results in noise by noise source location (noise from the left, or NH ear side with green diamonds; noise from the front with orange squares; noise from the right, or hearing impaired ear side with purple diamonds).

Figure 4 shows performance at the three test intervals for the Adaptive SRT test. A lower score indicates better performance. Panel A shows P1’s performance in quiet. The softest level that P1 was consistently able to identify target speech from a closed-set of four spondees in quiet did not differ substantially by test interval. Her SRT in quiet ranged from 12.9 dB pre-surgery to 10.3 dB at 3 months post-surgery. This is comparable to performance of a group of 24 NH adults (ages 22–67 years) on this same task (see Figure 6 of Firszt et al., 2012b). Panel B shows performance in the presence of the three noise types, female talker (green diamonds), male talker (orange squares) and MTB (purple triangles). Performance was more difficult with MTB than with each single-talker noise type and results were very similar in the presence of female and male talker noise. Performance improved at each successive test interval for each noise type except with female talker noise. Performance with MTB showed the greatest improvement over time (pre-surgery SRT = 48.4 dB, 3-month SRT = 45.6 dB and 9-month SRT = 44.0 dB). Panel C shows results by noise location: right side with hearing loss (purple triangles), front (orange squares) and left side with the intact ear (green diamonds). The task was easier with noise from the affected side (right) than noise from the front (orange squares) or from the intact ear side (left). P1’s scores for noise from the front were similar to scores for noise from the intact ear side.

**Figure 5:**
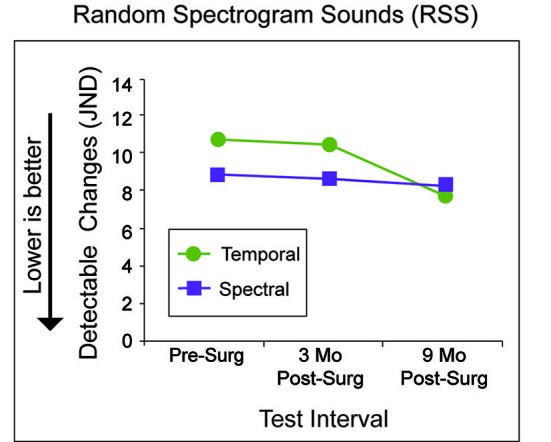
**P1’s minimum detectable changes (JND; *y*-axis) for temporal (green circles) and spectral complexity (purple squares) are indicated for each test interval (*x*-axis).** Lower JNDs reflect better performance.

Figure 5 shows results from the two psychoacoustic measures with RSS stimuli that differed in spectral or temporal complexity with test interval along the *x*-axis and JND score along the *y*-axis. Performance for detecting differences from a standard of 16 spectral regions in the number of regions (purple squares) included in spectral RSS stimuli (e.g., spectral complexity) was very similar at all three test intervals (JND 8.8 pre-surgery to 8.2 at 9 months). P1’s performance detecting differences from a standard temporal rate of 30 Hz in the rates (green circles) of temporal RSS stimuli (e.g., temporal complexity) was similar at pre-surgery (JND 10.8) and at 3 months (JND 10.4). By 9 months P1’s JND for RSS temporal complexity differences had improved to 7.8. P1’s performance on both tasks is similar to that of a group of 20 NH adults, ages 23–62 years, listening bilaterally across four test runs (Figure 5; Firszt et al., 2012b).

**Figure 6:**
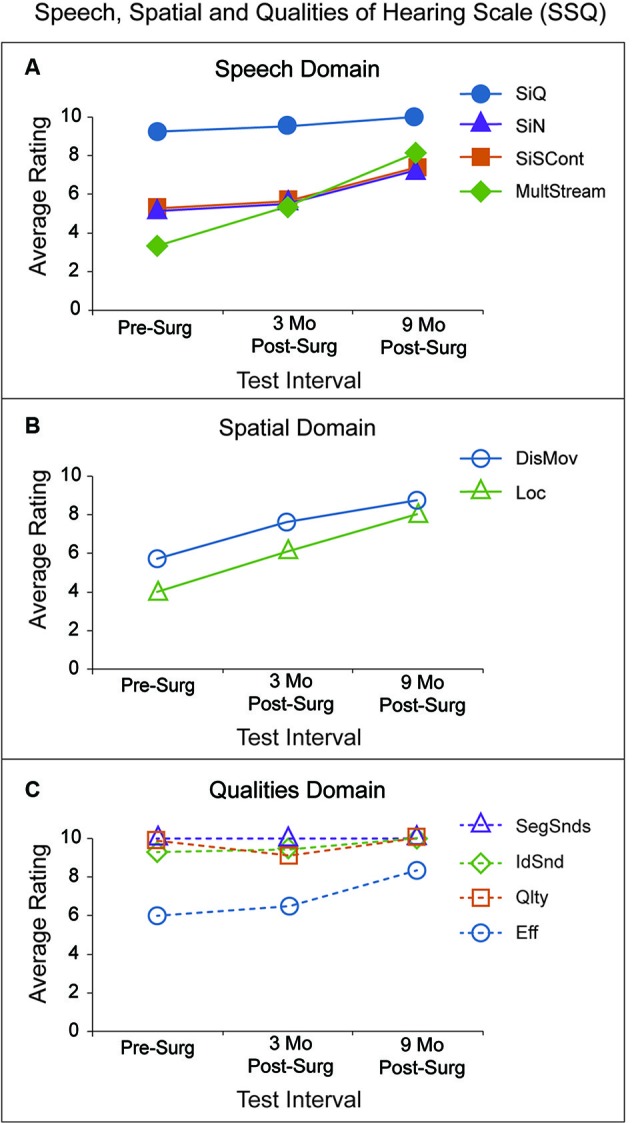
**SSQ ratings (*y*-axis) for 10 subscales are divided into three domains and plotted for each test interval (*x*-axis).** Panel **A** (Speech Domain) has P1’s average ratings for questions assessing four subscale areas: Speech in Quiet (SiQ; blue circles), Speech in Noise (SiN; purple triangles), Speech in Speech Contexts (SiSCont; orange squares), and Multiple Speech Stream Processing and Switching (MultStream; green diamonds). P1’s average ratings for questions assessing the two subscales within the Spatial Domain are shown in Panel **B**: Distance and Movement (DisMov; purple circles) and Localization (Loc; green triangles). Average subscale ratings within the Qualities Domain are indicated in Panel **C**: Segregation of Sounds (SegSnds; purple triangles), Identification of Sound and Objects (IdSnd; green diamonds), Sound Quality and Naturalness (Qlty; orange squares) and Listening Effort (Eff; purple circles).

Figure 6 shows SSQ questionnaire results describing self-perceived abilities in various listening scenarios by domain and subscale. The rating plots in Panel A are from four subscales in the Speech domain: Speech in Quiet (SiQ) as blue circles, Speech in Noise (SiN) as purple triangles, Speech in Speech Contexts (SiSCont) as orange squares, and Multiple Speech Stream Processing and Switching (MultStream) as green diamonds. Mean and SD subscale ratings by a group of 21 NH adults, ages 27–73, were reported by Dwyer et al. (2013). For reference, P1’s ratings are compared to that NH group’s ratings. P1’s SiQ ratings were similar to the NH group’s ratings at all three test intervals (9.3 pre-surgery to 10 at 9 months). P1’s SiN ratings improved over time (5.1 pre-surgery to 7.3 at 9 months), but were poorer than the NH group. The SiSCont and MultStream ratings improved over time and were similar to the NH group’s ratings by 9 months post-surgery (SiSCont 5.3 pre-surgery to 7.4 at 9 months; MultStream 3.3 pre-surgery to 8.2 at 9 months).

The rating plots in Panel B are from two subscales in the Spatial domain: Distance and Movement (DisMov) as purple circles and Localization (Loc) as green triangles. P1’s ratings for both subscales improved over time and were similar to the NH group ratings by 9 months (DisMov 5.7 pre-surgery to 8.7 at 9 months; Loc 4.0 pre-surgery to 8.0 at 9 months). The rating plots in Panel C are from four subscales in the Qualities domain: Segregation of Sounds (SegSnds) as purple triangles, Identification of Sound and Objects (IdSnd) as green diamonds, Sound Quality and Naturalness (Qlty) as orange squares and Listening Effort (Eff) as purple circles. SegSnds, IdSnd and Qlty ratings were all above nine and comparable to the NH group ratings at all three test intervals. Pre-surgery, Eff was poorer than that of the NH group but improved and was similar to that group’s ratings by 9 months (6.0 pre-surgery to 8.3 at 9 months). In summary, P1’s ratings were poorer than ratings of the NH adults reported by Dwyer et al. (2013) on 6 of 10 subscales prior to surgery. Each of those subscales improved and was more similar to the NH group’s ratings by the 9-month test interval except for the SiN subscale.

**Figure 7:**
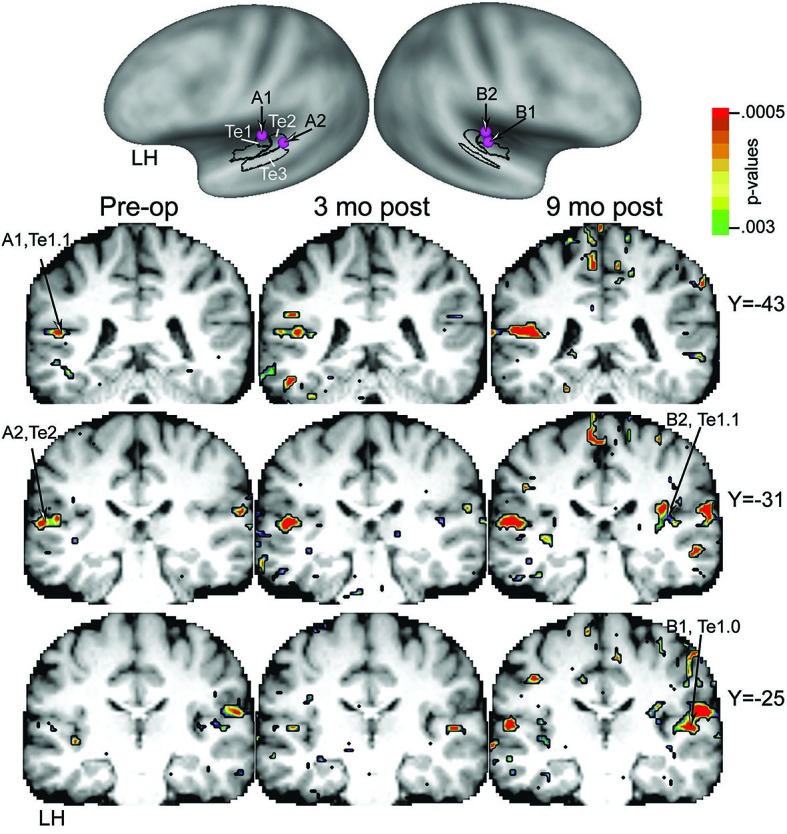
**Top row shows superior temporal plane on inflated left and right hemispheres.** Black borders indicate the schematic position of auditory cortical fields Te1 to Te3. Labeled purple spheres are placed over cortical surface location of peak *F*-*Z*stats in activated cortex. Bottom columns of coronal slices through P1’s structural brain images at the level of the superior temporal plane coincident with Te1 to Te3 auditory cortex. Left to right columns of identical slices show significantly activated cortex, respectively, at test intervals pre-surgery, 3, and 9 months post-surgery. Sites labeled A1, A2, B1, and B2 indicate corresponding locations on the cortical surface and coronal sections.

The top row of Figure 7 shows an inflated and laterally tilted view of the PALS-B12 atlas that reveals auditory cortex on the superior temporal plane. Drawn onto this plane are cytoarchitectonic borders for three auditory cortical fields (Te1, Te2, and Te3) (Glasser and Van Essen, 2011). The labeled purple spheres (A1, A2, B1 and B2) and comparable locations on the coronal sections indicate the locations of regions of interest strongly activated by the RSS stimuli (i.e., foci with peak *F*-*Z*stats). The most affected auditory cortical fields were Te1 and Te2. As shown in the left-most column of coronal sections prior to surgery, the extent of significant activation was limited, bilaterally distributed, but slightly more extensive in the left hemisphere (LH), ipsilateral to the stimulated unaffected left ear. The activated zone was larger bilaterally at 3 months post surgery as shown in the middle column of sections. Activity was even more widely distributed by 9 months as shown in the right-most column of sections. Especially prominent at 9 months was a substantial response distribution contralateral to the stimulated left ear that extended over most of the posterior superior temporal plane and spread more than 1 cm from anterior to posterior. The affected contralateral cortex included all Te auditory cortical fields at 9 months post surgery.

**Figure 8:**
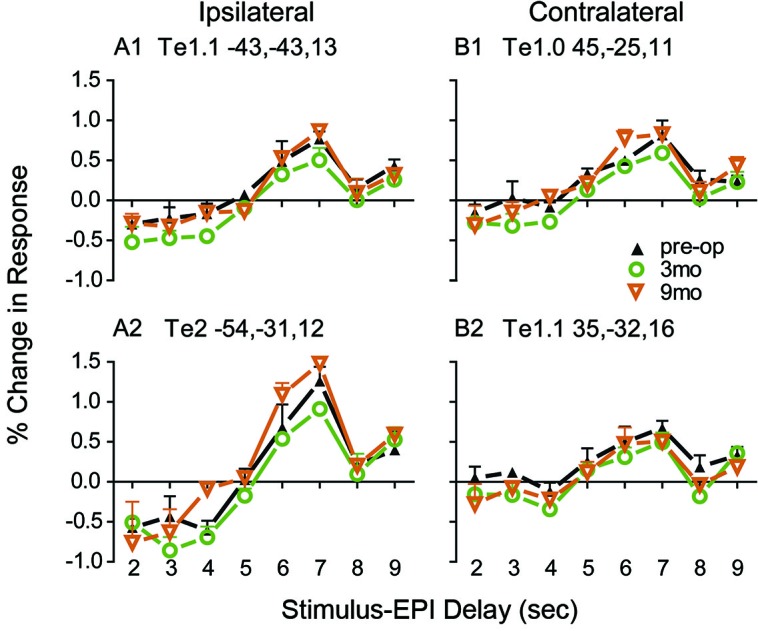
**Reconstructed BOLD response time courses based on RSS delays of 2–9 seconds during silent periods immediately before the next volume acquisition.** Plotted at each stimulus-EPI delay is the mean and standard error for the per cent change in BOLD amplitude over baseline from trials with no stimulation. BOLD % change response amplitudes extracted from 24 trials per stimulus-EPI delay and averaged across the voxels in spherical ROI with 5 mm radii. A different sphere was centered on the Talairach coordinates listed in each of the four panels. Separately colored connected lines and symbols show time courses for test intervals pre-surgery, 3, and 9 months post-surgery.

The panels in Figure 8 show response time courses from core (Te1.0 and Te1.1) and posterior belt (Te2) auditory fields. Response amplitudes were largest at 9 months post- surgery, especially in the belt area Te2 ipsilateral to the stimulated left ear and also in the anterior core Te1.0 field, contralateral to the left ear. Amplitudes were more nearly equal bilaterally before and after surgery in the center of primary auditory cortex located in Te1.1 fields of each hemisphere.

## Discussion

Corrective surgery for the conductive portion of the hearing loss substantially improved hearing thresholds from 0.25 to 6 kHz. Prior to surgery, P1 could not detect sound in the mid frequencies and perceived loud sounds as soft in the lowest and highest frequencies of the affected right ear. After surgery, air-conduction thresholds were close to P1’s bone-conduction thresholds, closing the air-bone gap. Sensorineural threshold levels remained, as expected, and thus hearing levels were essentially in the mild to moderately-impaired range, reaching 25–35 dB HL at 0.25, 3, 4 and 6 kHz.

Source localization significantly improved following surgery. The largest improvement was between pre-surgery and the 3-month post-surgery test interval with stable performance between the 3 and 9-month test intervals. Wilmington et al. (1994) also showed significant localization improvements pre to post-operatively when measured at 4 and 24 weeks after surgery to correct congenital UCHL in a group of patients’ ages 6–33 years; however, only three of the individuals were within the authors’ normal confidence interval after hearing correction. Similar to our individual case, there were no significant changes in localization performance over time post-operatively for their study participants; that is between the 4 and 24-week test intervals.

Considering speech recognition in noise, P1’s pre-surgical abilities varied by measure as did whether performance improved post-surgery. For example, on the *R*-Space task, P1’s ability to understand sentences in the presence of restaurant noise improved between the pre-surgery and 9-month post-surgery test intervals. Noise type and location affected Adaptive SRT results. P1’s SRT scores were similar when listening to words in the presence of single talker noise regardless of talker gender. P1’s poorest SRTs were in the presence of MTB noise, as were the greatest improvements. With respect to the noise source, it was not surprising that P1 was able to hear the words at softer levels when noise was towards the affected (right) ear, even after surgery, than when the noise was towards the unaffected (left) ear or from the front. These results for the affected and unaffected ear were similar to Wilmington’s (1994) results using a speech-in-noise task with low and high context sentences and competing babble (revised SPIN test, Bilger et al., 1984). When noise was toward the atretic ear, the majority of participants were within their NH group’s 95% confidence interval, but when noise was toward the NH ear, only three of the 14 participants were within the NH confidence interval. A reported case of corrected long-term UCHL (Anderson, 1985) also had more difficulty understanding speech in speech-shaped noise than 10 NH controls through 5 months post-correction, even at an advantageous SNR (+20 dB). The reduced performance was present for both the affected and unaffected ears. By 14 months post-correction, performance matched that of the NH control group at positive SNRs but continued to be poorer than NH controls at 0 dB SNR, a fairly common SNR in noisy environments.

For the psychoacoustic task using RSS stimuli, results showed no change in detecting spectral complexity differences after surgical correction of hearing to the right ear. RSS temporal complexity detection did improve at the 9-month test interval, however performance at all test intervals (including pre-operative) was similar to that of NH individuals listening bilaterally (Firszt et al., 2012b). Thus, detection of spectral and temporal cues was not enhanced by unilaterally improved acoustic hearing for this participant. Perhaps this task is less reliant on binaural processing than listening in noise or localizing sound sources. It should be noted that for this task and the speech recognition measures, P1 completed the measures at three test intervals, introducing the possibility of a training effect, whereas the NH group data provided for comparison were from single test sessions.

Results of the SSQ were in general consistent with findings of the behavioral measures. For four of the subscales (SiQ and all of the subscales in the Qualities Domain except Eff), P1’s ratings were between 9–10 and similar to ratings of the NH group (*n* = 21) reported by Dwyer et al. (2013). For all other subscales except SiN, ratings were poorer than those of Dwyer’s NH group pre-surgery but more similar after 9 months binaural hearing experience. P1 continued to have more difficulty than the NH group, even after 9 months experience with SiN.

The self-assessment results from the SSQ were augmented by comments from P1. According to the participant, 1 week after surgery, all sounds were louder, at times exceptionally loud and even startling; leading to the need to depart from the immediate environment when this occurred and find a location that was quiet. Strategies for dealing with noise that had worked in the past, such as turning the impaired ear towards the noise source, no longer worked and led to confusion. Hearing from behind was a completely new sensation, and also created uncertainty as to where sound was coming from. P1 was involved with music, gave piano and violin lessons, and performed in a band. Sound quality and pitch changes were common during the early post-operative period. It was not always clear “what to make of all this sound”.

Three months after surgery, sounds and sources were less confusing, sound was not as loud, and localization and sound quality had improved. P1 reported hearing new sounds that were not previously audible, even though P1 had one NH ear. The ability to hear at greater distances was notable, as was the ability to find and identify sounds in the surroundings without being able to see them. Prior to surgery, P1 often was unaware of a sound until the corresponding source came into view. This changed after surgery, when for example, hearing a person before seeing them was described as something novel. Being in noisy environments was less distracting and the ability to overhear conversation while holding a different conversation was a new experience. P1 commented that while regaining hearing abilities in the right ear, there was a sensation that the left ear (the unaffected ear) was also changing at the same time, and the two ears were “starting to play nice together”. The result was hearing in stereo, again a notion that P1 had not realized prior to hearing correction. Finally, P1 reported increased feelings of confidence and reduction of effort to hear and communicate in everyday compared to pre-surgery. When questioned 9 months after surgery, the comments from P1 were similar to what had been previously reported. Overall, the descriptions mimic a period of over excitation of sound followed by adaptation and shifting to allow both ears to contribute to improved hearing performance using enhanced binaural abilities.

A case study presented by Stange et al. (2001) was of a 62 year old woman with a congenital maximum UCHL who reported hyperacusis (abnormal sensitivity to sound intensity) for more than 2 years following hearing restoration. Both the case reported by Stange et al. (2001) and the current case involved congenital onset and extensive time with UCHL and both reported an increased sensitivity to sound. However in the current case, the sensitivity diminished within the first 3 months following hearing restoration. One possible explanation for the difference may be that P1 continued to have some sensorineural hearing loss after surgery. Prior to surgery, P1 demonstrated a large asymmetry between ears; however hearing thresholds were 50–70 dB HL at 3 kHz and above, providing some high frequency input. Although this hearing was not considered useful based on participant report, some stimulation was present and may have contributed to quicker assimilation of the new auditory information. In this case, extended asymmetry in hearing did not prevent auditory system changes consequent to restored binaural hearing. Also promising was that P1 made significant improvements in binaural abilities despite hearing correction in adulthood at age 41. Gray et al. (2009) reported on combined data from two different studies (participant’s age range 6–53 years, mean pre-operative hearing loss about 64 dB) and suggested that after surgery for aural atresia, older (38 years and greater) congenital adults performed more poorly when noise was toward the corrected ear than younger adults. Assessments were made, however, only 1 month after hearing correction and participant ages were not provided.

There was also evidence of neuroplastic reorganization in auditory cortex that especially involved core and belt regions in the current case. The overall extent and amplitudes of contralateral auditory cortex responses increased after hearing recovery, resulting in increased bilateral activation. Vasama et al. (1994) also found enhancements in auditory cortex in six participants with congenital UCHL using magnetoencephalography (MEG). They showed the expected asymmetric larger amplitudes in five patients and shorter latencies in three patients over the hemisphere contralateral to the stimulated NH ear (Vasama et al., 1994). The six patients ranged in age from 7–28 years and had mean hearing threshold levels in the affected ear near 70 dB HL. Only one patient showed larger amplitudes over the ipsilateral hemisphere, a finding similar to reports of cortical responses in patients with profound unilateral sensorineural hearing loss (Khosla et al., 2003; Hanss et al., 2009; Burton et al., 2012, 2013; Maslin et al., 2013). Of note, this individual also had greater hearing loss (about 90 dB HL). A later study compared pre-operative and 2-months post-operative auditory-evoked magnetic fields in seven adults ages 26–51 with UCHL who underwent surgery for otosclerosis or abnormal ossicular chains (Vasama et al., 1998). Duration of hearing loss was 6–14 years and hearing loss in the affected ears was 57 dB HL before surgery and 17 dB afterwards. Based on MEG recordings, all patients showed cortical response changes after surgery and correction of the UCHL. Changes took the form of earlier latencies and increased response strengths contralateral to the stimulated ear, with some ipsilateral changes. Although mean pre-operative values did not differ from NH controls, the post-operative changes differed. This finding parallels that of our present participant, suggesting modification to cortical responses following hearing improvement.

In summary, a re-established balance to binaural activity might explain the trend toward improved performance on measures after the stapedotomy procedure for the studied participant. The SSQ results showed that ratings of the participant’s perceived ability were similar to the NH comparison group (Dwyer et al., 2013) by 9 months on all subscales except SiN. It is possible that both the pre-surgery unilateral auditory deprivation and the post-surgery continued asymmetry between ears (due to the sensorineural hearing loss in the affected ear) influenced the speech perception in noise deficits. Persistent sensorineural hearing loss might also explain retained symmetrical activation of auditory cortex as opposed to a normally asymmetrical contralateral activation pattern in NH.

The improvements noted in binaural abilities were clinically promising, because they indicated that auditory system connections could re-activate with elimination of conductive hearing problems despite being physiologically latent well beyond any imagined critical developmental period. Most encouraging was that the restored physiology in native connectivity, even if only partial, had substantial behavioral consequences for the participant. The mechanisms responsible for these changes are unknown. However, they might arise from increased balance to binaural neural firing rates with the reintroduction of crossed inhibition that lead to changes in central gain mechanisms. At the same post-surgery test intervals, the observed enhanced contralateral activity in core and adjacent auditory cortical fields possibly are a manifestation of these changes in central gain. In contrast, sudden sensorineural unilateral hearing loss following surgical resections of acoustic neuromas result in the opposite effect of reduced crossed inhibition from the affected ear together with possible reduced central gain (Burton et al., 2013; Maslin et al., 2013). Together, these results support the need for future research of unilateral auditory deprivation effects and plasticity, with consideration for length of deprivation, age at hearing correction, degree of hearing, and type of hearing loss (conductive versus sensorineural).

## Conflict of interest statement

The Associate Editor, Dr. Jonathan Peelle declares that, despite being affiliated to the same institution as the authors (the Department of Otolaryngology of Washington University School of Medicine), handled the review process objectively and no conflict of interest exists.
